# Glutamine-glutamate centered metabolism as the potential therapeutic target against Japanese encephalitis virus-induced encephalitis

**DOI:** 10.1186/s13578-024-01340-3

**Published:** 2025-01-22

**Authors:** Mengyuan Li, Hang Yuan, Xiaofei Yang, Yingfeng Lei, Jianqi Lian

**Affiliations:** 1 Department of Infectious Diseases, Tangdu Hospital, Air Force Medical University, Xi’an, 710038 China; 2 Pathogenic Biology, Medical College of Yan’an University, Yan’an, 716000 China; 3 Department of Microbiology, School of Basic Medicine, Air Force Medical University, Xi’an, 710032 China

**Keywords:** Japanese encephalitis virus, Metabolism, Viral encephalitis

## Abstract

**Background:**

Japanese encephalitis (JE) induced by Japanese encephalitis virus (JEV) infection is the most prevalent diagnosed epidemic viral encephalitis globally. The underlying pathological mechanisms remain largely unknown. Given that viruses are obligate intracellular parasites, cellular metabolic reprogramming triggered by viral infection is intricately related to the establishment of infection and progression of disease. Therefore, uncovering and manipulating the metabolic reprogramming that underlies viral infection will help elucidate the pathogenic mechanisms and develop novel therapeutic strategies.

**Methods:**

Metabolomics analysis was performed to comprehensively delineate the metabolic profiles in JEV-infected mice brains and neurons. Metabolic flux analysis, quantitative real-time PCR, western blotting and fluorescence immunohistochemistry were utilized to describe detailed glutamine-glutamate metabolic profiles during JEV infection. Exogenous addition of metabolites and associated compounds and RNA interference were employed to manipulate glutamine-glutamate metabolism to clarify its effects on viral replication. The survival rate, severity of neuroinflammation, and levels of viral replication were assessed to determine the efficacy of glutamine supplementation in JEV-challenged mice.

**Results:**

Here, we have delineated a novel perspective on the pathogenesis of JE by identifying an aberrant low flux in glutamine-glutamate metabolism both in vivo and in vitro, which was critical in the establishment of JEV infection and progression of JE. The perturbed glutamine-glutamate metabolism induced neurotransmitter imbalance and created an immune-inhibitory state with increased gamma-aminobutyric acid/glutamate ratio, thus facilitating efficient viral replication both in JEV-infected neurons and the brain of JEV-infected mice. In addition, viral infection restrained the utilization of glutamine via the glutamate-α-ketoglutaric acid axis in neurons, thus avoiding the adverse effects of glutamine oxidation on viral propagation. As the conversion of glutamine to glutamate was inhibited after JEV infection, the metabolism of glutathione (GSH) was simultaneously impaired, exacerbating oxidative stress in JEV-infected neurons and mice brains and promoting the progression of JE. Importantly, the supplementation of glutamine in vivo alleviated the intracranial inflammation and enhanced the survival of JEV-challenged mice.

**Conclusion:**

Altogether, our study highlights an aberrant glutamine-glutamate metabolism during JEV infection and unveils how this facilitates viral replication and promotes JE progression. Manipulation of these metabolic alterations may potentially be exploited to develop therapeutic approaches for JEV infection.

**Graphical Abstract:**

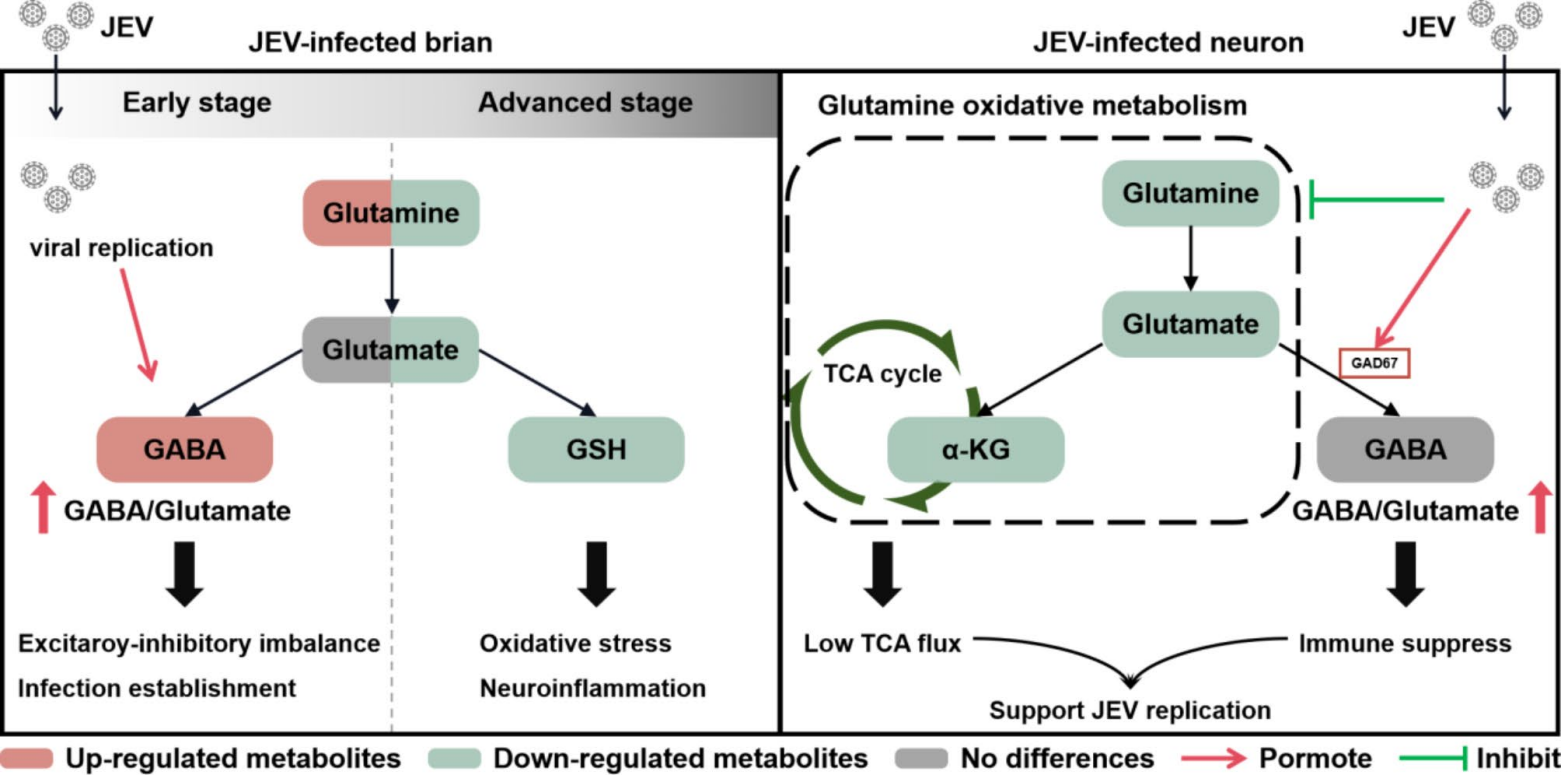

**Supplementary Information:**

The online version contains supplementary material available at 10.1186/s13578-024-01340-3.

## Introduction

Japanese encephalitis virus (JEV) is a mosquito-transmitted flavivirus that primarily affects children and occasionally affects adults. Japanese encephalitis (JE) induced by JEV infection is the leading global cause of viral encephalitis with symptoms ranging from mild fever to severe encephalitis and even death. There are almost 68,000 global cases of JE annually according to World Health Organization (WHO). One in 4 cases is fatal and half of the survivors develop permanent neurological or psychiatric sequelae [1]. JEV is highly neuroinvasive, and once it breaches the blood-brain barrier (BBB), the virus spreads rapidly in the central nervous system (CNS), resulting in severe neuroinflammation and ultimately neuronal death [2]. The lack of specific treatment after the occurrence of JE is the major cause of high morbidity and mortality. Thus, effective antiviral and anti-inflammatory treatments are urgently required for patients with JE.

Since viruses are obligate intracellular parasites, reprogramming host cell metabolism for viral protein synthesis, genome replication and viral particle production is a common strategy to complete their life cycle [3]. In the context of viral infection, the metabolism of glucose and glutamine, as the metabolic hubs of multiple biological processes that include the biosynthesis of energy, lipids, amino acids and nucleotides, are exploited by pathogens through diverse strategies [4]. For glucose metabolism, a common consequence is to induce preferentially utilization of glucose through glycolysis rather than tricarboxylic acid (TCA) cycle (referred as Warburg effect) under aerobic conditions [5, 6]. Increased glycolysis, lactate production and glucose uptake are the typical metabolic changes associated with viral infection. DNA viruses, such as Adenovirus, upregulate glycolysis for optimal replication, with viral E4ORF1 binding to MYC to boost glycolytic gene expression [7]. RNA viruses, including SARS-CoV-2 [8], Rhinovirus [9], Hepatitis C Virus [10], and Dengue virus [11], also enhance glycolysis post-infection. For example, upregulated glycolytic intermediates and the expression of key enzymes were described both in human hepatoma (Huh7) cells infected with HCV [12] and endothelial cells infected with Dengue virus [11]. While increased glycolytic activity frequently facilitates viral replication, the activation of the TCA cycle often tends to be detrimental to viral propagation [13]. TCA metabolites and their derivatives, including aconitate, succinate and fumarate, often act as anti-inflammatory, anti-oxidant, and anti-microbial molecules to impede viral replication, such as Zika virus (ZIKV) [14] and SARS-Cov-2 [15].

For glutamine metabolism, several viruses, such as human immunodeficiency virus HIV [16] and KSHV [17] can drive the catabolism of glutamine via glutaminolysis to replenish the TCA cycle and support viral infection. Furthermore, some oncogenic viruses like HCV can also promote glutamine utilization by producing antioxidant glutathione, whose antioxidant activity alleviates virus-induced oxidative stress and maintains persistent infection [18]. In a recent study using a murine norovirus model, the authors found that the viral NS1/2 protein is critical for enhancing glutaminase activity and upregulating glutaminolysis, which is essential for efficient viral replication [19]. Thus, different viruses employ diverse strategies to hijack host cell metabolism. Infection is an orchestrated interaction between pathogen and host. Therefore, manipulating the metabolic reprogramming underlying the virus-induced infection is a promising approach to influence the outcome of infection [4, 20].

Here, we performed metabolomics analysis to comprehensively characterize the metabolic profiles of mice brains at different stages of encephalitis induced by JEV infection. In vivo data revealed the extensive reshaping of the glutamine-glutamate-centered metabolism during the onset and progression of JE. Consistently, JEV infection of neurons induced similar reprogramming of glutamine-glutamate-related metabolism to maintain optimal viral replication in vitro. In this study, we thoroughly characterized the altered glutamine-glutamate-related metabolism during JEV infection, and unveiled how this phenomenon benefits viruses. Meanwhile, our data also indicated that manipulating glutamine-glutamate metabolism is a promising therapeutic approach for JEV infection.

## Materials and methods

### Cells and viruses

The mouse neuroblastoma cells Neuro2a were cultured in Dulbecco’s Modified Eagle’s Medium (DMEM, Procell, Cat. No. PM150210) containing 10% (v/v) fetal bovine serum (FBS, Gibco, Cat. No. 16140071) and 1% Penicillin-Streptomycin Solution (P/S, Procell, Cat. No. PB180120), incubated at 37℃ in a humidified atmosphere with 5% CO_2_.

C6/36 *Aedes albopictus* cells used for virus propagation were grown in Minimum Essential Medium (MEM, Procell, Cat. No. PM150410) containing 10% FBS and 1% P/S for virus propagation. C6/36 cells were incubated at 28℃ in a humidified atmosphere of 5% CO_2_. The JEV wild-type strain P3 was obtained from our laboratory. Virus stocks were produced by collecting cell-free supernatant from infected C6/36 cell culture after 4 days post of infection at multiplicity of infection (MOI) of 0.01. Viral purification and titer determination were described previously [21].

### Compounds

The 200mM L-Glutamine stock solution was purchased from Procell Life Science & Technology (Cat. No. PB180420). Glutamic acid (Glutamate, Sigma-Aldrich, Cat. No. G1251) was dissolved in 1 M HCl. The pH was adjusted with NaOH to 7.2 to make a 1 M stock solution of glutamate, which was sterilized by filtration through a 0.2 µM filter. γ-Aminobutyric acid (GABA) purchased from Topscience (Cat. No. T0508) was dissolved in sterile water to make a 50mM stock solution. Dimethyl 2-oxoglutarate (7 M), a cell-permeable α-ketoglutaric acid (α-KG) analog, was obtained from Sigma-Aldrich(Cat. No. 349631). The glucose transport inhibitor phloretin (Cat. No. T2924) was obtained from Topscience and diluted in accordance with the instructions.

### Viral infection and treatment with compounds

Neuro2a cells were seeded in 6-well or 12-well plates 12 h before infection to produce an 80% confluence monolayer. Cells were infected with Mock (heat-inactivated virus suspension) or JEV at MOI of 0.1 for 2 h in serum-free DMEM. After adsorption, the inoculum was discarded, and the cells were washed twice with phosphate-buffered saline(PBS, Procell, Cat. No. PB180327). Subsequently, Neuro2a cells were incubated in fresh DMEM containing 2% FBS and 1% P/S at 37℃ in a humidified atmosphere of 5% CO_2_. For compound treatment experiments, JEV-infected Neuro2a cells were treated with different compounds or equal volumes of specific compound dilution agents at 37℃ for 24 h.

### Animal experiment

All animal experiments were reviewed and approved by the Animal Experimental Welfare & Ethical Inspection of the Laboratory Animal Center, Air Force Medical University (No.20230610).

For metabolomics analysis and immunofluorescence assay, 4-week-old female C57BL/6 mice were infected with viable or heat-inactivated JEV (1 × 10^6^ pfu) by footpad injection. The behavioral scores of infected mice were recorded daily at certain time. Scoring criteria were described previously [22]. Briefly, mice with mild weight loss, tremor, piloerection and behavioral scores of 2–3 were considered as Early JE (generally at 4 dpi). Mice with significantly weight loss, severe convulsions and behavioral scores of 4–5 were considered as Advanced JE (generally at 5 dpi). It should be emphasized that the survival time following JEV infection varies due to individual differences among the mice. Therefore, during the sampling process, we first determined Day 4 as the early stage of JE. Then, behavioral scoring was conducted every 6 hours in Day 4 and sampled the mice with early JE symptoms in batches rather than operating uniformly at a fixed time. Similar sampling was also performed for mice with advanced encephalitis.

For glutamine supplementation experiments, 4-week-old female C57BL/6 mice were infected with viable or heat-inactivated JEV (1 × 10^6^ pfu) by footpad injection. Glutamine (250 mg/Kg) or equivalent PBS were administrated daily via intraperitoneal injection (ip) for five consecutive days after JEV infection.

### Sample preparation for metabolomics analysis

For cellular Metabolomics analysis, 1 × 10^7^ Neuro2a cells were seeded in 15 cm cell culture dishes overnight. The infection protocol was performed as described above. At 24 h post-infection, the cells were washed and scraped off the dishes for subsequent measurement. Six biological replicates in Mock- or JEV-infected Neuro2a cells were set up for cell Metabolomics analysis.

For metabolomics analysis of brain tissues, mice were anesthetized and transcardially perfused with cold PBS. Mice brains from Mock, Early JE and Advanced JE groups were harvested and immediately snap-frozen in liquid nitrogen and stored at -80℃ for measurement. Ten biological replicates were established for each group.

### Metabolites extraction, liquid chromatography-mass spectrometry (LC-MS/MS) analyses and data processing

Extraction of metabolites and LC-MS/MS analyses of Neuro2a cells and brain tissues of mice were performed by Biotree Biotech (Shanghai, China).

For the metabolic data analysis of brain tissues, *N* = 30 combined with R^2^X(cum) = 0.554 were set as model parameters for principal component analysis. Mock-2 and Advanced JE-5 that exceeded the 95% confidence interval (Hotelling’s T-squared ellipse) were excluded from the subsequent analysis. Metabolites with the variable importance in the projection (VIP) of the first principal component of the OPLS-DA model>1 and of Student’s *t*-test *p*<0.05 were considered as significantly changed metabolites. Differential metabolites with an MS2 score>0.9 were included in heatmap analysis (https://www.bioinformatics.com.cn). Kyoto Encyclopedia of Genes and Genomes (KEGG) enrichment analysis of significantly changed metabolites were performed and the top 10 pathways with smallest raw *p*-value between pairwise groups were screened for presentation. For the analysis of specific metabolites, the maximum and minimum values of each group were excluded to reduce the within-group variation.

### Metabolic flux analysis

Glutamine labeled with ^13^C ([U-^13^C_5_] glutamine) was obtained from Cambridge Isotope Laboratories (Cat. No.184161-19-1) and added to glutamine-deprived DMEM (Procell, Cat. No.PM150213). DMEM containing 4mM [U-^13^C_5_] glutamine, 10% FBS, and 1% P/S was used for cell culture.

Neuro2a cells were seeded in a 6-well plate overnight before infection to produce a 80% confluence monolayer. Then, cells were Mock infected (heat-inactivated virus suspension) or infected with JEV at an MOI of 0.1 for 2 h in serum free DMEM. After adsorption, the cells were washed twice with PBS and covered with prepared medium. Cells were harvested at 5 min and 2 h post-infection according to the instructions provided by metabolic flux analysis platform (Phenions, Shanghai, China).

### Quantitative real-time PCR

Total RNA were extracted with Total RNA Kit 1 (Omega Bio-Tek, Cat. No.R6834-02). The concentration of the extracted RNA was measured with a NanoDrop One spectrophotometer (ThermoFisher). cDNA was synthesized using Hifair^®^ II 1st Strand cDNA Synthesis SuperMix (Yeasen, Cat. No.11120ES60). Quantitative real-time PCR (qRT-PCR) was performed with Hieff^®^ qPCR SYBR Green Master Mix (Yeasen, Cat. No.11201ES03) according to the manufacturer’s protocol. The mRNA expression level of target genes was normalized with *β-actin*. The qRT-PCR primers are listed in Table S1.

### Western blotting

Cells were lysed with RIPA Buffer (ThermoFisher, Cat. No.89901) containing Protein Phosphatase Inhibitor (Solarbio, Cat. No.P1260). Western blot was performed following the standard method. The primary antibodies were: anti-JEV NS3 (GeneTex, Cat. No.GTX638821), anti-β-Tubulin (Proteintech, Cat. No.10068-1-AP), anti-β-Actin (Proteintech, Cat. No.66009-1-Ig), anti-GLS (Proteintech, Cat. No.12855-1-AP), anti-GLUD1 (Proteintech, Cat. No.14299-1-AP), anti-OGDH (Proteintech, Cat. No.15212-1-AP). The secondary antibodies used in Western blotting were IRDye^®^ 800CW goat anti-rabbit IgG (Abcam, Cat. No.ab216773) and Alexa Fluor^®^ 680 donkey anti-mouse IgG (Abcam, Cat. No.ab175774).

### Immunohistochemistry and hematoxylin and eosin (H&E) staining

Mice were anesthetized and transcardially perfused with cold PBS followed by 4% paraformaldehyde (PFA, Beyotime, Cat. No.P0099). Brains were post-fixed with 4% PFA overnight. Subsequent steps including tissue sections preparation, staining, microscope inspection and image acquisition were provided by Servicebio (Wuhan, China). Multiplex immunofluorescence staining is based on TSA (tyramide signal amplification) staining technology. The key to this technique is the ability to perform multiple rounds of staining without concern for cross-reactivity between antibodies from different rounds, as the tyramide reaction is independent of the antibody binding events.

The following primary antibodies were used: anti-JEV NS3 (GeneTex, Cat. No.GTX638821), anti-GABA (Sigma-Aldrich, Cat. No.A2052), anti-L-Glutamate (Abcam, Cat. No.ab9440). Nuclei were stained with Hoechst (Sangon Biotech, Cat. No.E607302).

### Immunofluorescence assays

Cells were washed 3 times with PBST and fixed with ice-cold 4% PFA for 15 min. Then, 0.1% Triton X-100 was added for 15 min at room temperature. Subsequent blocking and staining were performed according to the manufacturer’s instruction using the Cy3 Immunofluorescence Detection Kit (Sangon Biotech, Cat. No.E670002). Nuclei were stained with Hoechst (Sangon Biotech, Cat. No.E607302). The primary antibody was anti-JEV NS3 (GeneTex, Cat. No.GTX638821).

### Small interfering RNA (si-RNA) transfection

The sequences of siRNA targeting GLUD1 and OGDH were designed by Sangon Biotech (Shanghai, China). The siRNA sequences are shown in Table S1. The siRNA transfection was performed with jetPRIME^®^ transfection reagent (Polyplus, Cat. No.101000046) at 24 h prior to JEV infection according to the manufacture’s protocol.

### Enzymatic activity of OGDH

JEV-infected Neuro2a cells that were untreated or treated with 250µM glutamate or 7mM α-KG for 2 h were washed 3 times with PBS and harvested for enzymatic activity analysis. The determination was performed using the Dehydrogenase (α-KGDH) Activity Assay Kit (Solarbio, Cat. No.BC0715).

### Plaque-forming assay

The BHK-21 cells were plated into 12-well tissue culture plates and grown to 80% confluence. The cells were washed twice with PBS to remove any debris. Subsequently, the cells were infected with a series of tenfold dilutions of the virus-containing samples and incubated at 37 °C for 2 h to facilitate viral adsorption. Then, cell monolayer was covered with overlay medium comprised of 25 mL 4×DMEM, 25 mL ddH_2_O, 50 mL 4% methycellulose and 2 mL FBS, and incubated at 37℃ in a humidified atmosphere of 5% CO_2_. After 4 to 5 days, the overlay medium was removed and the cell monolayer was stained with 1% crystal violet.

### Statistical analyses

Except for the special statistical analysis of metabolomic data described above, other statistical analysis of data from mice and cell experiments utilized GraphPad Prism version 8.1 (GraphPad, CA). Differences between two groups were assessed by two-tailed unpaired Student’s *t*-test. A *p*-value<0.05 was considered significant (*p<0.05, **p<0.01, and ***p<0.001).

## Results

### Overview of mice brain metabolic landscape during the progression of JE

**Fig. 1:**
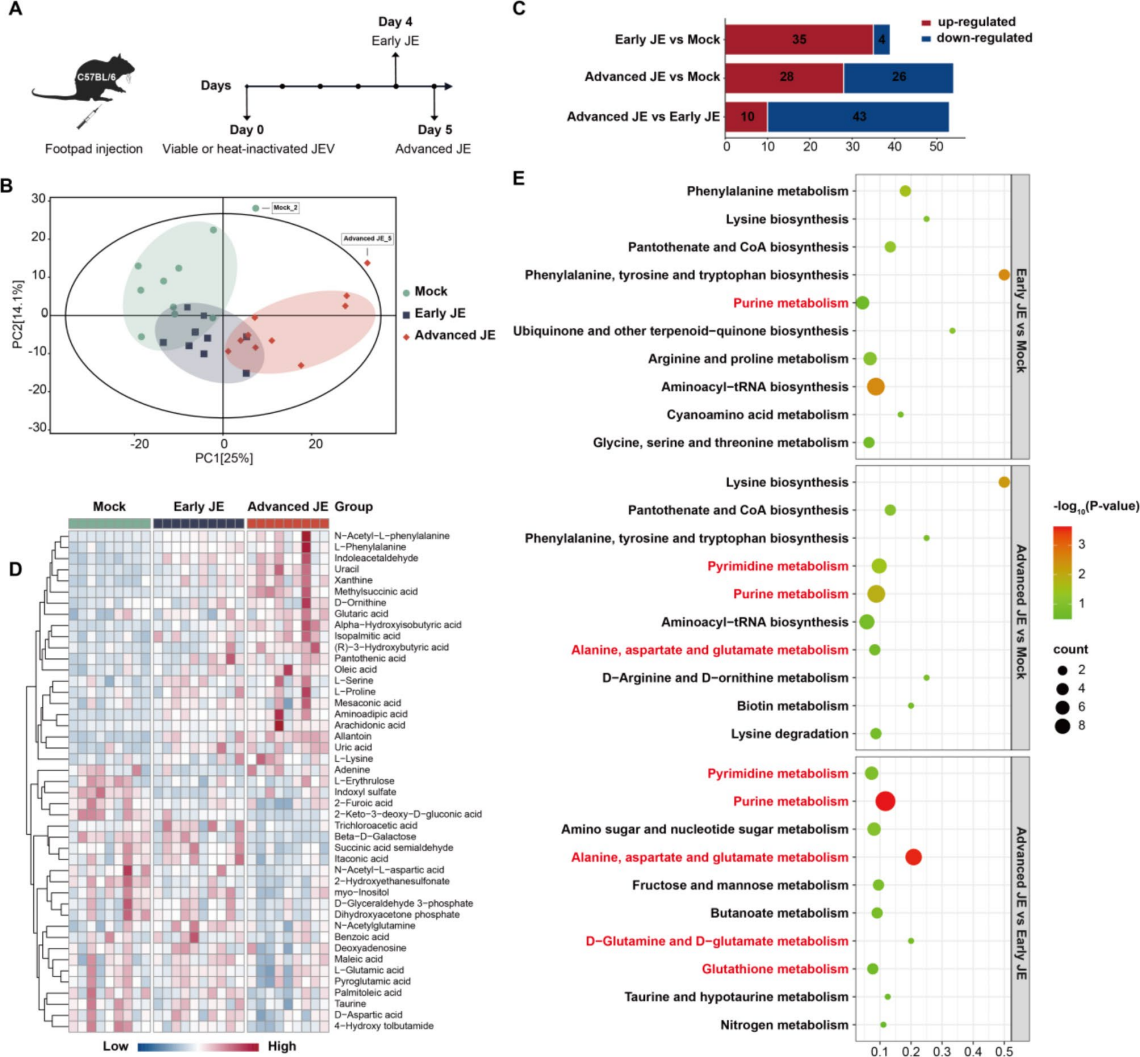
Global metabolomic profiling of mice brain tissues during the progression of JE. **A** Schematic representation of JEV infection and mice brains collection at different time points post of infection. **B** Principal component analysis (PCA) of untargeted metabolomics of mice brains in Mock, early JE and advanced JE groups. Each point represents a single sample and is separated by color and shape. Mock-2 and Advanced JE-5 were outside the 95% confidence interval (Hotelling’s T-squared ellipse) and were excluded from subsequent analyses. **C** Stacked bar chart of differentially expressed metabolites with MS2 name-matched between pairwise groups (Early JE vs. Mock, Advanced JE vs. Mock and Advanced JE vs. Early JE). **D** Cluster heatmap analysis of all differentially expressed metabolites identified between pairwise groups (Early JE vs. Mock, Advanced JE vs. Mock and Advanced JE vs. Early JE, MS2 name matched and MS2 score＞0.9) **E** Top 10 KEGG pathways based on pathway enrichment analysis among Early JE vs. Mock, Advanced JE vs. Mock and Advanced JE vs. Early JE groups (top 10 pathways with the smallest *p*-value)

To obtain the overview metabolic profiles in different stages of encephalitis induced by JEV infection, mice brain tissues were harvested at day 4 post-infection (Early JE, characterized by mild weight loss, tremor and piloerection) and day 5 post-infection (Advanced JE, mouse with significantly weight loss and severe convulsions) for untargeted metabolomics analysis (Fig. 1A). Metabolomics data was acquired by MS/MS (tandem mass spectrometry). Sample molecules were ionized and measured in the first mass spectrometer (MS1). Selected ions were then fragmented to generate characteristic fragment ions for further analysis in the second mass spectrometer (MS2). Then, an in-house MS/MS database (BiotreeDB) was applied in metabolite annotation, which have been widely used in many metabolic studies [23]. After obtaining original metabolomic data, principal component analysis (PCA, Fig. 1B) and the permutation test of supervised Orthogonal Projections to Latent Structures Discriminant Analysis (OPLS-DA, Supplementary Fig. 1) were employed to delineate metabolic distinctions between Mock, Early JE and Advanced JE groups. As shown in Fig. 1B, the metabolic profiles of the samples in the three groups were well distinguished with more concentrated dots within groups and more separated dots between groups. The Early JE group was positioned between the Mock and Advanced JE groups in PCA plots, reflecting its transitional metabolic state. To sum up, untargeted global metabolomics based on LC-MS/MS identified a total of 7045 metabolites (negative ion mode), of which 169 matched with the MS2 name. Pairwise comparisons were performed between the Mock, Early JE, and Advanced JE groups. Differentially expressed metabolites were identified by combining the Variable Importance in the Projection (VIP) value (>1) in the OPLS-DA model and *p*-value<0.05 in the Student’s *t*-test, and then presented as volcano plots (Supplementary Fig. 1).

The number of differentially expressed metabolites with matched MS2 names between pairwise groups were enumerated. The results are presented as stacked bar graph in Fig. 1C. Comparison of the Mock and Early JE groups revealed that 35 metabolites were up-regulated and 4 metabolites were down-regulated. Comparison between the Advanced JE and Mock groups revealed that 28 metabolites were up-regulated and 26 metabolites were down-regulated. Notably, the comparison between the Advanced JE and Early JE groups showed a predominance of down-regulated metabolites, with only 10 metabolites being up-regulated. These metabolic features exhibited by Advanced JE resembled a “post-war silent” status in which the synthetic functions of brain cells were heavily impaired, leading to intracranial depletion of metabolites.

In order to make the original data more intuitive, heatmap analysis was employed to visualize all MS2 name matched differential metabolites (differentially expressed in any pairwise comparison) with its MS2 spectral match score>0.9. As shown in Fig. 1D, the metabolic landscape in the brains of mice was substantially altered as JE progressed. Subsequently, differential metabolites were annotated and assigned for KEGG pathway enrichment analysis. The top 10 KEGG pathways with the lowest *p*-value were selected and displayed (Fig. 1E). The metabolism of amino acids and nucleotides were mainly enriched in the KEGG analysis. Notably, several pathways related to glutamine-glutamate metabolism were also significantly enriched, which are intricately linked to nucleotides and amino acids metabolism, indicating its crucial role in JEV infection.

### JE driven intracranial glutamine-glutamate metabolism reprogramming

**Fig. 2:**
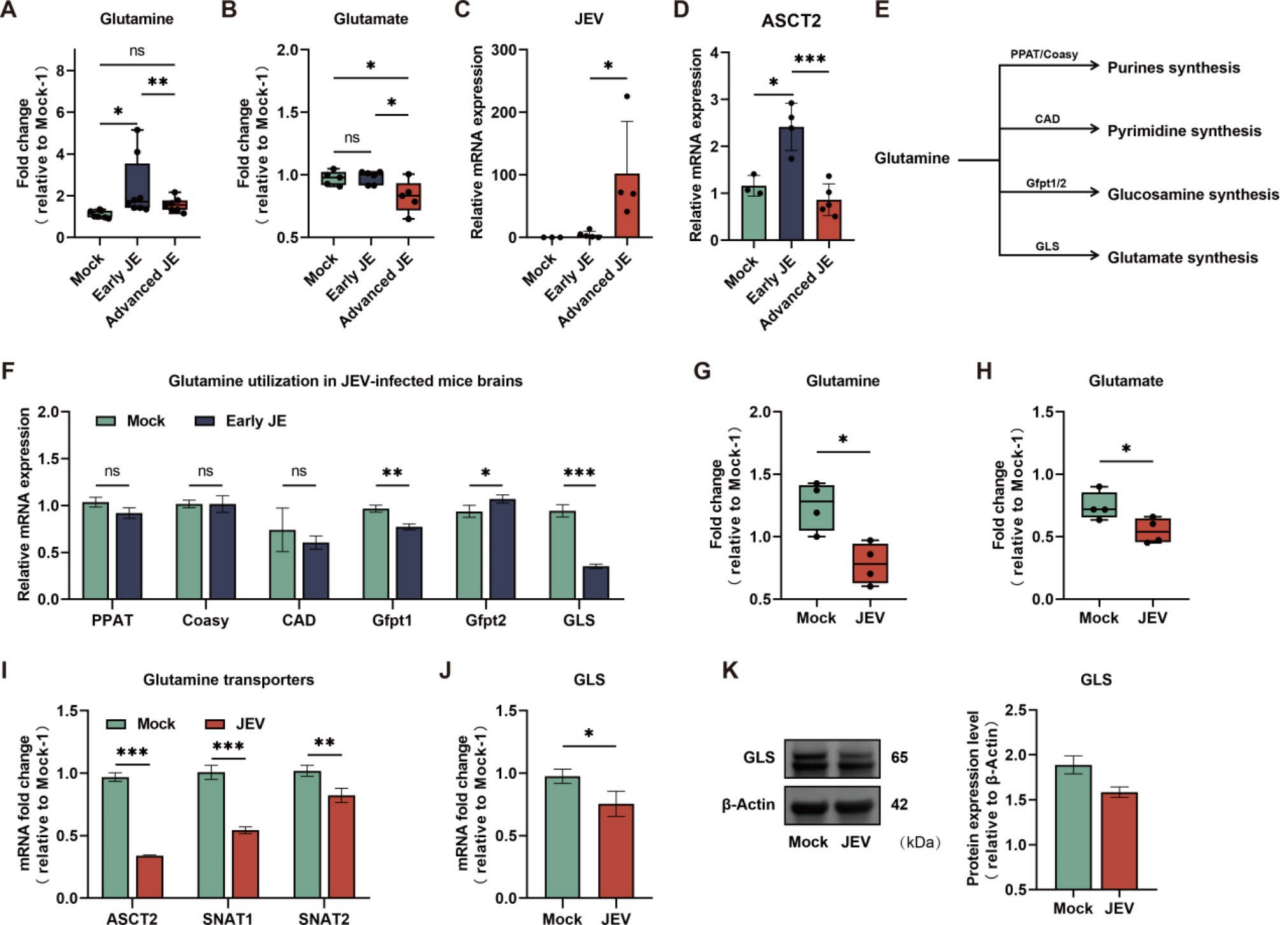
Metabolomics analysis reveals aberrant reduction in glutamine-glutamate metabolism during JEV infection. **A** Levels of glutamine in mice brain during the development of JEV-induced encephalitis. **B** Levels of glutamate in mice brain during the development of JEV-induced encephalitis. **C** mRNA level of JEV in mice brains during the development of JE. **D** mRNA level of ASCT2 in mice brains during the development of JE. **E** Simplified schematic representation of key enzymes of glutamine utilization. **F** The mRNA levels of genes involved in glutamine utilization in Mock- and Early-JE groups. **G** and **H** Levels of glutamine and glutamate detected in Mock and JEV infected Neuro2a cells at 24 hpi. **I** The mRNA levels of glutamine transporters in Mock- and JEV-infected Neuro2a cells at 24 hpi. **J** and **K** The mRNA levels and protein levels of GLS in Mock- and JEV-infected Neuro2a cells at 24 hpi

Given the findings of significant changes in glutamine-glutamate related metabolism in the KEGG enrichment analysis, we further investigated the levels of glutamine and glutamate. As shown in Fig. 2A, the level of glutamine was elevated in the early stage of JE and then, decreased as JE progressed. However, as a principal metabolic product of glutamine, glutamate was not elevated in Early JE in response to the increased glutamine levels. Instead, it sharply declined with the reduction of glutamine in Advanced JE (Fig. 2B). By assessing the expression levels of metabolic genes involved in glutamine uptake and utilization, we observed a significant up-regulation of the glutamine transporter ASCT2 in Early JE. However, as viral replication intensified and JE progressed, its level decreased in the Advanced JE group (Fig. 2C and D). However, the actual utilization of glutamine in critical bio-synthetic pathways, such as purine (PPAT and Coasy), pyrimidine (CAD) and glucosamine (Gfpt1/2), did not increase correspondingly with elevated glutamine levels in Early JE (Fig. 2E and F). In particular, a notable reduction was observed in the level of glutaminase (GLS), an enzyme responsible for catalyzing glutamine to glutamate. This might account for the absence of increased glutamate levels in Early JE despite a significant increase in the precursor glutamine.

Given that intracranial glutamine levels are closely related to the metabolic activity of glutamine in astrocytes, we also investigated the glutamine metabolic profiles of the murine astrocyte cell line C8-D1A during JEV infection. As shown in Fig S2A, there was a significant enhancement in glutamine uptake and utilization at 24 hpi. However, by 48 hpi, metabolic activity declined with intensive viral replication. This in vitro result aligns with our metabolomics data, indicating that the level of glutamine was initially increased with enhanced uptake but decreased in the advanced stages of infection.

Since participating in the generation of reduced glutathione (GSH), a predominant intracellular antioxidant and redox regulator, is the critical biological role of glutamine and glutamate at metabolic facet [24]. We found that the levels of GSH in the brains of JEV-infected mice were notably reduced and even depleted in the Advanced JE (Fig S2B). Although the levels of its oxidized form, GSSG, did not significantly change, the GSH/GSSG ratio was markedly reduced upon JEV infection. As a reliable indicator for oxidative imbalance suggested by previous studies, this pronounced reduction in the GSH/GSSG ratio indicated intensive intracranial oxidative stress during JE. We further examined key genes involved in GSH metabolism in the brains of JEV-infected mice. As shown in Fig S2C, GSH metabolism was also impaired after JEV infection, suggesting that the low flux of glutamine-glutamate metabolism is an important metabolic factor contributing to the disruption of redox balance during JE.

Since JEV is a highly neurotropic virus capable of efficient viral replication in neurons, characterizing the glutamine-glutamate associated metabolic profiles in JEV-infected neurons is necessary to clarify the aberrant glutamine-glutamate metabolism exhibited in the mouse brain. Hence, the mouse neuroblastoma cell line Neuro2a infected with JEV were employed for untargeted metabolomics analysis. As shown in Fig. 2G and H, the levels of glutamine and glutamate were markedly reduced in JEV-infected neurons. We next analyzed the mRNA expression levels of glutamine transporters ASCT2, SNAT1 and SNAT2 in JEV-infected neurons to determine whether the reduction of glutamine levels was due to decreased uptake. As depicted in Fig. 2I, a significant reduction in glutamine uptake in JEV-infected neurons was evident. Furthermore, the expression level of GLS was also suppressed by JEV infection, resulting in a decreased conversion of glutamine to glutamate (Fig. 2J and K). Given the reduction in metabolic precursors, the levels of neuronal GSH and GSSG also correspondingly decreased. While the GSH/GSSG ratio showed no significant difference, there was still a noticeable decline in JEV-infected Neuro2a cells (Fig S2D). Although the expression levels of key enzymes in GSH metabolism were up-regulated in virus-infected neurons (Fig S2E), the lack of necessary metabolic precursors impeded the effective restoration of GSH levels. These findings are consistent with previous studies that the disabled GSH metabolism and imbalanced redox are major factors in the pathogenesis of JE [20, 25].

Collectively, these results highlight an aberrant reduction in glutamine-glutamate metabolism upon JEV infection both in vivo and in vitro.

### JEV infection inhibits neuronal utilization of glutamine via the TCA cycle in early stage of viral replication

**Fig. 3:**
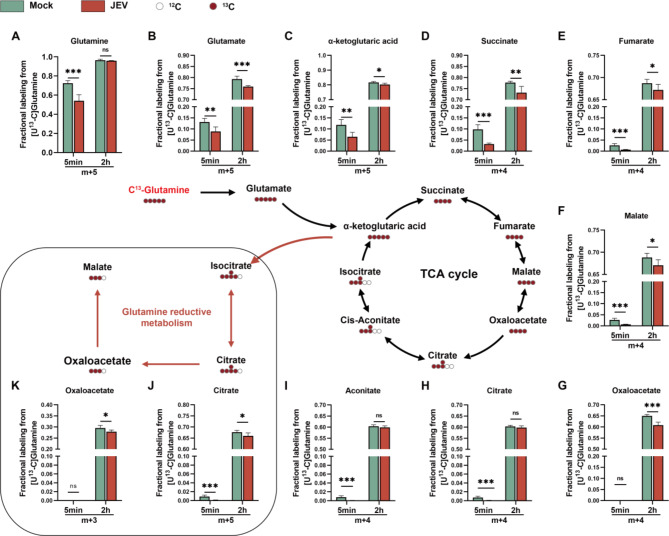
JEV infection inhibits cellular utilization of glutamine via TCA cycle in Neuro2a cells. Glutamine metabolic flux analysis in JEV-infected Neuro2a cell line. Schematic representation of ^13^C5-labelled glutamine metabolic tracing. Isotope-labeled carbon is indicated by red circle and unlabeled carbon as colorless circle. After attachment of virions to Neuro2a cells, the cell culture medium was replaced with [U-^13^C_5_] glutamine-contained fresh medium for subsequent incubation. Cells were further cultured for 5 min and 2 h, and then collected for metabolic flux analysis. Bar charts show the fraction of intermediate metabolites produced by ^13^C5-labelled glutamine in mock or JEV infected Neuro2a cell line at 5 min and 2 h post-infection. Glutamine reductive metabolism shown in the rectangular box, and oxidative metabolism in outside. **A** Glutamine; **B** Glutamate; **C** α-ketoglutaric acid; **D** Succinate; **E** Fumarate; **F** Malate; **G** Oxaloacetate; **H** Citrate; **I** Aconitate; **J** and **K** Citrate and Oxaloacetate in glutamine reductive metabolism

To further elucidate the characteristics of neuronal glutamine metabolism upon JEV infection, 13 C-labeled glutamine ([U-^13^C_5_] glutamine) metabolic flux analysis was performed in JEV-infected Neuro2a cells. As displayed in Fig. 3A, the fraction of labeled glutamine was significantly reduced (Mock, 72.4%; JEV, 54%) at the early stage of JEV infection (5 min). However, the proportion of intracellular labeled glutamine in JEV-infected Neuro2a cells was comparable to that of the Mock group at 2 h post-infection. We further extended the infection time to specifically address whether JEV infection in neurons affects glutamine uptake. As determined by the absolute quantity of isotope labeled glutamine at different timepoints after viral infection, we found that the cellular uptake of glutamine was consistently reduced, except for 2 h post-infection (Fig S3A). The findings are consistent with the decreased level of intracellular glutamine in the metabolomics data of JEV-infected Neuro2a cells described above, suggesting that the glutamine uptake was suppressed by viral infection.

Subsequently, the proportions of glutamine-derived metabolites in the TCA cycle were analyzed to elucidate the contribution of glutamine in fueling oxidative phosphorylation. The fractions of these glutamine-derived metabolites were generally decreased in Neuro2a cells infected with JEV at 5 min and 2 h post-infection. In particular, the fractions of glutamate (Fig. 3B), α-ketoglutaric acid (α-KG, Fig. 3C) and succinate (Fig. 3D), which are more direct metabolites downstream of glutamine oxidative metabolism, were notably reduced after JEV infection. In addition, the rectangular box displayed in Fig. 3 highlights the significant inhibition of glutamine reductive metabolism, which suggested by the lower fractions of m + 5 citrate (Fig. 3J) and m + 3 oxaloacetate (Fig. 3K) in JEV-infected Neuro2a cells.

Given that both glutamine and glucose act as physiological energy substrates in neurons that can fuel the TCA cycle, we next investigated whether glucose is more likely to be utilized than glutamine in JEV-infected neurons. Determination of the expression levels of glucose transporters in JEV-infected Neuro2a cells (Fig S3B) revealed that the glucose uptake might be increased with the up-regulation of the sodium dependent glucose transporter 1(SGLT1) rather than the more ubiquitously expressed glucose transporter 3 and 4 (GLUT3 and GLUT4) in neurons. We further conducted glucose deprivation and anaplerosis to determine whether JEV replication in neurons is glucose-dependent. As Fig S3C shown, the absence of glucose induced the suppression of viral replication, while the levels of viral replication in Neuro2a cells were restored upon glucose re-supplementation Moreover, phloretin, which competitively inhibits SGLTs, was employed to block glucose uptake in JEV-infected neurons. The results in Fig S3D and S3E indicated that the inhibition of SGLT-mediated glucose uptake also restrains JEV replication.

The collective data reveal a glucose-dependent, rather than glutamine-dependent metabolic pattern in JEV replication. This metabolic reprogramming strategy aligns with the common consequence of viral infection, which is to induce highly elevated glucose metabolism, causing aerobic glycolysis rather than oxidative phosphorylation through the TCA cycle.

### Glutamine-glutamate-α-KG metabolic axis drives TCA cycle to inhibit JEV replication in neurons

**Fig. 4:**
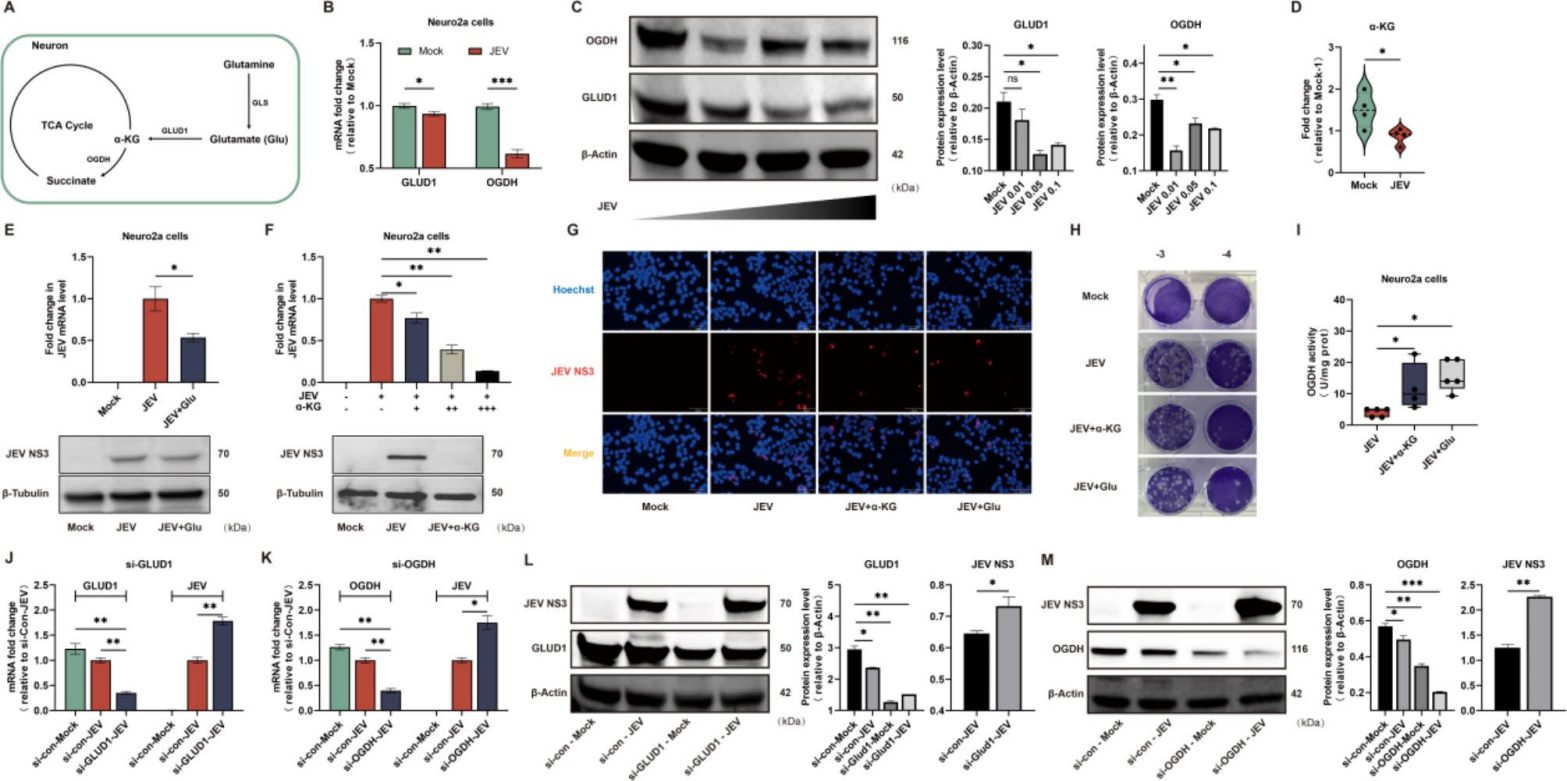
Glutamine-glutamate-α-KG axis drives TCA cycle to inhibit JEV replication in neurons. **A** Simplified schematic representation of oxidative glutamine metabolic pathway. **B** The mRNA levels of GLUD1 and OGDH in Mock- and JEV-infected Neuro2a cells at 24 hpi. **C** The protein levels of GLUD1 and OGDH in Mock- and JEV-infected Neuro2a cells (MOI = 0.01, 0.05, 0.1) at 24 hpi. **D** Level of α-KG detected in Mock and JEV infected Neuro2a cells at 24 hpi. **E** JEV mRNA levels and viral NS3 protein levels in Mock- and JEV-infected Neuro2a cells treated with 250µM glutamate addition in complete culture medium at 24 hpi were assessed by qPCR and Western blot. **F** JEV mRNA levels were measured in Mock- or JEV-infected Neuro2a cells with various concentrations of α-KG (1.75mM,3.5mM and 7mM) for 24 h, protein expression levels of JEV NS3 in Mock-infected, JEV-infected Neuro2a cells with or without 7mM α-KG-added were determined by Western blot. **G** Immunofluorescence analysis of JEV replication in Neuro2a cells. Nuclei stained with Hoechst (blue), JEV NS3 protein in red. α-KG: 7mM; Glu: 250µM. **H** Detection of infectious viral particles released in cell culture medium treated with 250µM glutamate or 7mM α-KG by plague forming assay. **I** Effect of different metabolites addition on OGDH enzyme activity in JEV-infected Neuro2a cells. **J and K** si-con-Mock indicates Neuro2a cells transfected with control siRNA and infected with heat-inactivated JEV. si-con-JEV indicates Neuro2a cells transfected with control siRNA and infected with JEV. si-GLUD1 and si-OGDH are Neuro2a cells transfected with siRNA-GLUD1 (20nM) and siRNA-OGDH (20nM), and then infected with JEV. The mRNA levels of viral genome, GLUD1 and OGDH were determined by qPCR at 24 hpi. **L** and **M** The protein levels of JEV NS3, GLUD1 and OGDH were determined in Neuro2a cells at 24 hpi by Western blot

Given the inhibition of glutamine uptake and utilization via the TCA cycle observed in JEV-infected Neuro2a cells, we asked whether glutamine oxidation could negatively affect JEV propagation in neurons. Firstly, the expression levels of key enzymes involved in glutamine oxidation were determined (Fig. 4A). As depicted in Fig. 4B and C, the expression of glutamate dehydrogenase (GLUD1) and α-Ketoglutarate Dehydrogenase (OGDH) were all suppressed in JEV-infected Neuro2a cells. Metabolomic data further indicated a reduction in α-KG level following JEV infection (Fig. 4D). These data confirm that the viral infection inhibits the utilization of glutamine via glutamate-α-KG axis. Moreover, exogenous addition of glutamate and α-KG served adverse effects on viral propagation as assessed by qPCR, immunoblotting (Fig. 4E and F), immunofluorescence(Fig. 4G) and plaque assay(Fig. 4H). In particular, the addition of α-KG, a direct intermediate metabolite of the TCA cycle, inhibited JEV replication in a dose-dependent manner.

Data from our group and others have highlighted the importance of high glycolytic and low TCA cycle activities for optimal viral replication [5, 26]. To validate whether the exogenous addition of glutamate and α-KG nourish the TCA cycle, the enzymatic activity of OGDH, the first rate-limiting enzyme of α-KG entry into the TCA cycle, was determined. As shown in Fig. 4I, the enzymatic activity of OGDH was profoundly elevated with the supplementation of glutamate and α-KG, indicating its potential inhibitory effect on viral replication mediated by the TCA cycle. To assess whether blocking the conversion of glutamate to α-KG or the oxidation of α-KG in the TCA cycle could affect JEV replication, selective siRNA-mediated suppression of GLUD1 and OGDH expression was performed to disturb the glutamate-α-KG metabolic axis. As expected, knockdown of either GLUD1 or OGDH in JEV-infected Neuro2a cells resulted in higher levels of viral propagation as assessed by qPCR (Fig. 4J and K) and immunoblotting(Fig. 4L and M).

Overall, these findings indicate that the metabolism of glutamine via the glutamate-α-KG axis drives the TCA cycle and adversely affects JEV replication in neurons. Thus, viral infection induces the low flux of glutamine-glutamate metabolism in neurons to support optimal propagation of JEV.

### Altered glutamate-GABA metabolic pattern favors JEV replication

**Fig. 5:**
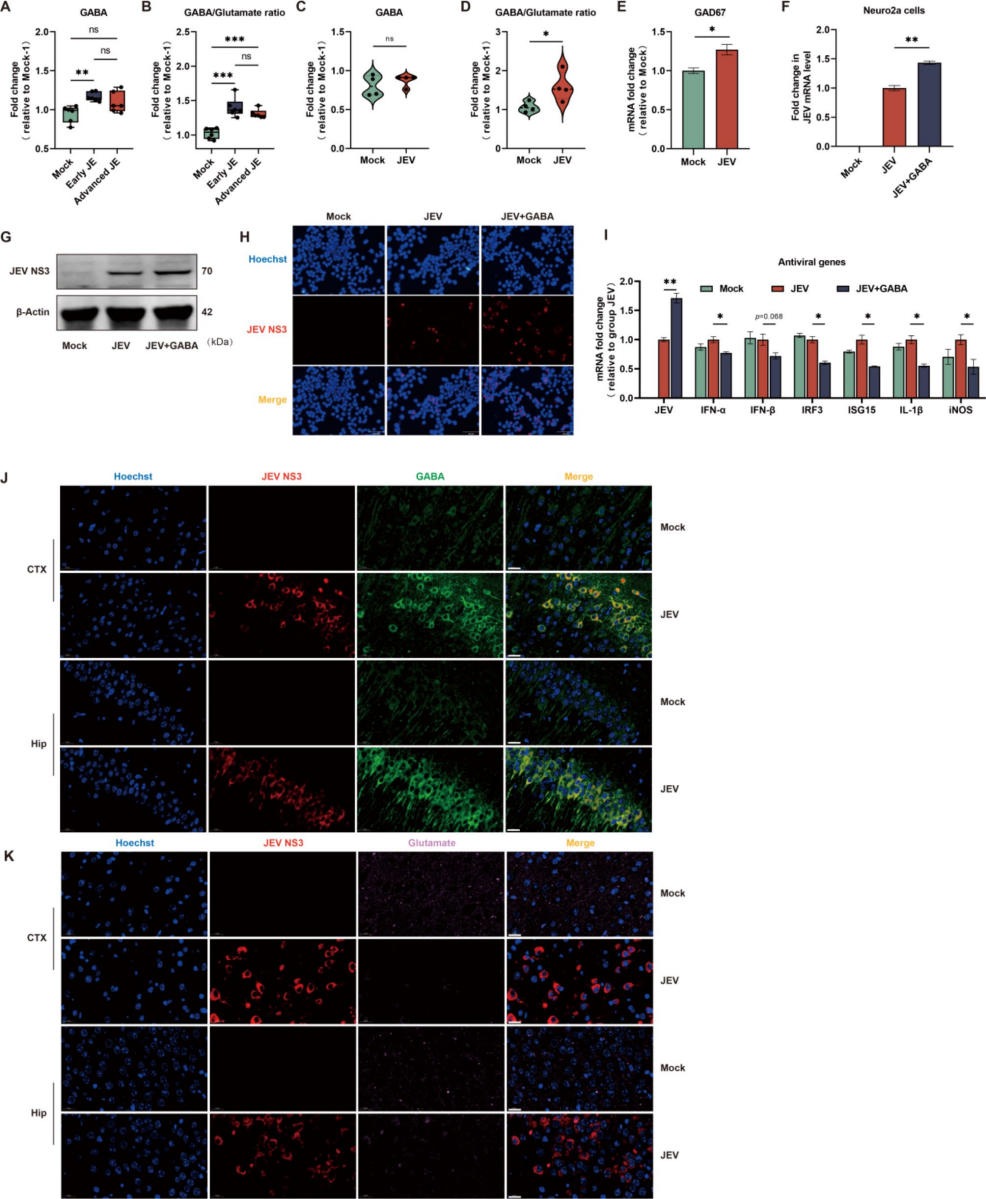
JEV infection induced altered Glutamate-GABA metabolic pattern both in neurons and mice brains. **A** Levels of GABA detected in mice brains from Mock, Early JE and Advanced JE groups. **B** Ratio of GABA and glutamate in Mock, Early JE and Advanced JE groups. **C** Levels of GABA detected in Mock and JEV infected Neuro2a cells at 24 hpi. **D** Ratio of GABA to Glutamate in Mock and JEV infected Neuro2a cells at 24 hpi. **E** The mRNA levels of GAD67 in Mock- and JEV-infected Neuro2a cells at 24 hpi. **F** and **G** JEV mRNA levels and viral NS3 protein levels in Mock- and JEV-infected Neuro2a cells treated with 100µM GABA addition in complete culture medium at 24 hpi were assessed by qPCR and Western blot. **H** Immunofluorescence analysis of JEV replication in Mock- and JEV-infected Neuro2a cells treated with 100µM GABA addition in complete culture medium at 24 hpi. Nuclei stained with Hoechst (blue), JEV NS3 protein in red. **I** mRNA levels of antiviral genes detected in Mock-, JEV-infected Neuro2a cells with 100µM GABA added. **J and K** Representive immunofluorescence microscopy images of Mock- and JEV infected mice brain at 4 day post of infection. CTX: cerebral cortex (cingulate cortex). Hip: Hippocampus (CA3). Scale bar = 20 μm. **J** Nuclei stained with Hoechst (blue), JEV NS3 in red, GABA in green. **K** Nuclei stained with Hoechst (blue), JEV NS3 in red and Glutamate in pink

In addition to conversion to α-KG for cellular energy supplement, glutamine and glutamate also act as the predominant metabolic precursors of GABA, the principal inhibitory neurotransmitter in the brain. Therefore, we further investigated whether abnormal glutamine-glutamate metabolism would affect neurotransmitter homeostasis during JE progression. As shown in Fig. 5A and B, the levels of GABA and the GABA/glutamate ratio in mouse brain were increased with the progression of encephalitis, suggesting an aberrant glutamate-GABA metabolism underlying JEV-induced encephalitis. Accordingly, we assessed whether altered glutamate-GABA metabolic profiles are presented in JEV-infected neuronal cells. First, the GABA content in Neuro2a cells infected with JEV was investigated via metabolomics analysis. Given the decreased level of glutamate (presented in Fig. 2H), the level of GABA, which is mainly derived from glutamate, should theoretically also be simultaneously reduced. However, the overall level of GABA was not significantly changed between Mock and JEV-infected neurons (Fig. 5C). Next, the ratio of GABA to glutamate was calculated to evaluate the inhibition-excitation state in neurons infected with JEV. As Fig. 5D shown, the GABA/glutamate ratio was also notably increased in JEV-infected group due to the reduced glutamate content, consistent with the metabolomic analysis from the brains of JEV-infected mice. Based on the low level of metabolic precursor glutamate, we next investigated whether viral infection promoted the generation of GABA to maintain its normal level. The expression level of glutamate decarboxylase 67 (GAD67, the major enzyme responsible for converting glutamate into GABA) was assessed. The mRNA level of GAD67 was increased after JEV infection, indicating that the conversion of glutamate to GABA was promoted (Fig. 5E).

To further investigate whether the elevated GABA/glutamate ratio benefits viral replication, JEV-infected Neuro2a cells were treated with exogenous addition of GABA. Consistent with our expectation, viral replication was significantly promoted in GABA-supplemented group as assessed by qPCR (Fig. 5F), immunoblotting(Fig. 5G) and immunofluorescence (Fig. 5H). Next, we assessed how GABA enhances virus replication. Increasing evidences indicates that neurotransmitters are critical modulators in neuro-immune crosstalk. Therefore, the expression level of cellular innate immunity-related genes were detected in Neuro2a cells treated with GABA. Although a potent antiviral response was not induced by JEV infection in neurons, the addition of GABA remarkably suppressed cellular innate immunity, as shown by decreased expression levels of antiviral-related genes including type 1 interferon, interferon regulatory factor 3 (IRF3), interferon-stimulated gene 15 (ISG15), interleukin-1β (IL-1β) and induced nitric oxide synthase (iNOS) (Fig. 5I).

In general, GABA acts as negative immune modulator and usually suppresses the cellular immune response [27, 28]. This immunosuppressive effect induced by GABA for tumor tolerance [29] and the establishment of viral infection [30] have been reported previously. Here, we provide evidence that GABA favors viral replication by restricting neuronal immunity. This provide a possible explanation for the maintenance of GABA level in JEV-infected neurons, even with reduced metabolic precursor glutamate.

As a high level of GABA is associated with encephalitis described in Fig. 5A, we next investigated the metabolic pattern of glutamate-GABA in vivo. Brain tissues from JEV-challenged mice at early JE stage were sampled for immunofluorescence. Consistent with the metabolomics analysis findings, increased GABA was observed in the brains of JEV-infected mice. Notably, virus-infected neurons (viral NS3 positive) exhibited extremely high GABA abundance either in the cerebral cortex or hippocampus (Fig. 5J). In contrast to this enrichment of GABA, a significant reduction in glutamate levels were observed in the infectious microenvironment (Fig. 5K). This finding is consistent with the observation in vitro that JEV infection preferentially promotes the conversion of glutamate to GABA, thereby facilitating viral proliferation.

The collective data indicate that infection by JEV induces a neuronal metabolic environment featuring a high level of GABA and low level of glutamate both in vitro and in vivo. This high GABA/glutamate ratio was accompanied by suppressed cellular innate immunity, thus favoring the establishment of viral infection.

### Glutamine supplementation alleviates encephalitis and improves survival in JEV-infected mice

**Fig. 6:**
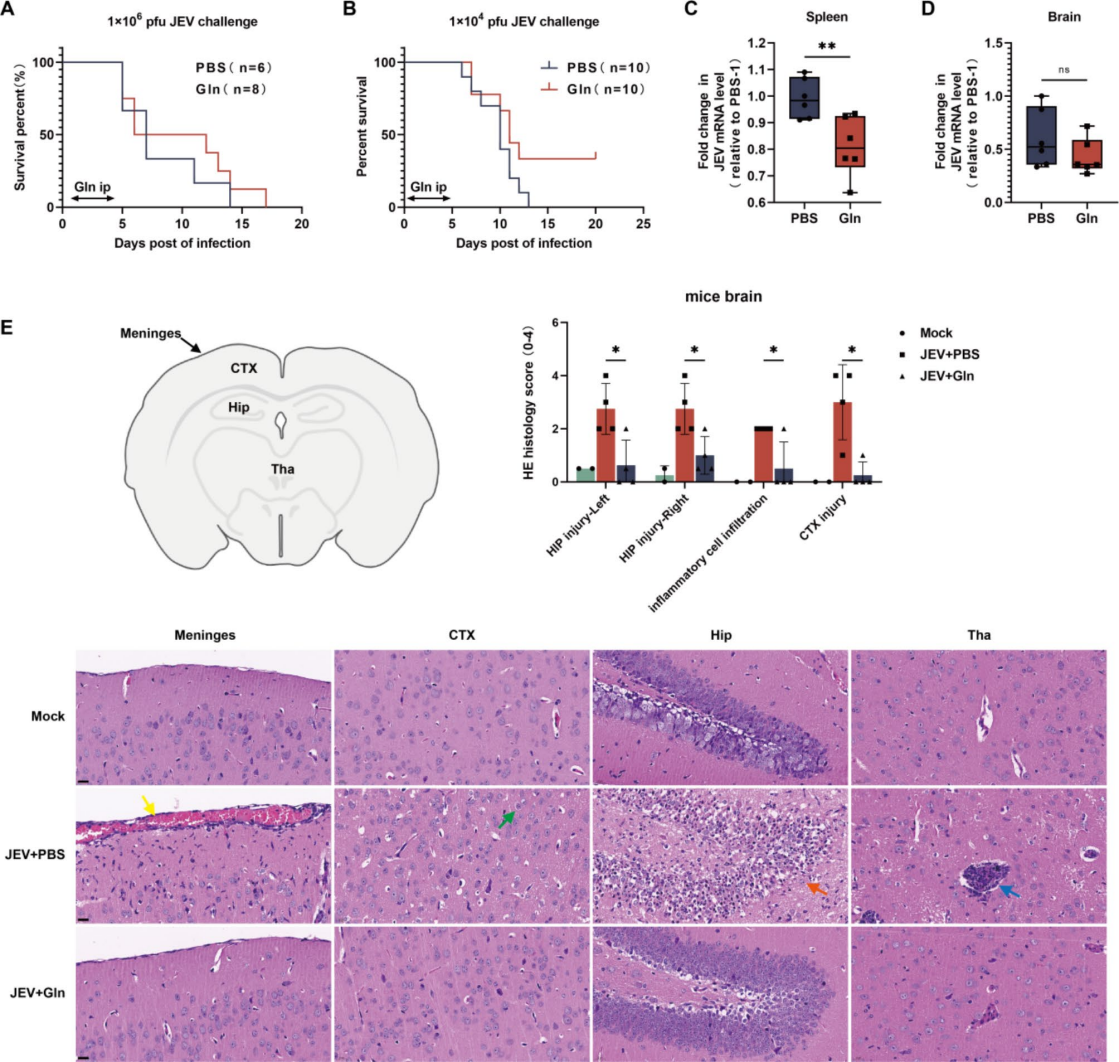
Glutamine supplementation enhances mice survival rate and alleviates encephalitis after JEV challenge. **A and B** Survival rate of JEV-infected mice administrated with PBS or Gln. Mice were administrated with 250 mg/Kg Gln or equivalent PBS via intraperitoneal injection (ip) for 5 consecutive days after JEV infection. **A**, High-dose JEV challenging. **B**, Low-dose JEV challenging. **C and D** Viral mRNA levels in the spleen tissues **(C)** and brain tissues **(D)** of mice infected with low-dose JEV treated with PBS and Gln at 5 days post-infection. **E** Brain tissues obtained from Mock-infected, low-dose JEV-infected mice treated with PBS or Gln were analyzed by hematoxylin and eosin (H&E) staining. Representative images of different section of mice brain (indicated in schematic diagram) and pathological scores analysis were employed to assess the severity of encephalitis caused by JEV infection. Yellow arrow: inflammatory cell infiltration and hemorrhage of the meninges. Green arrow: swollen neurons with pale-stained cytoplasm. Red arrow: necrosis of neurons accompanied with pyknotic and shrunken nuclei. Blue arrow: massive inflammatory cell infiltration. Scale bar = 20 μm

Based on the hints that glutamine-glutamate associated metabolism has a central role in JEV infection, we further evaluated the efficacy of glutamine supplementation in JEV-infected mice. Glutamine is the most abundant amino acids and has been used as a complementary treatment for several clinical conditions, such as diarrhea, cystic fibrosis, obesity, surgery, and injury [31]. In the present study, mice infected with JEV were intraperitoneally injected with 250 mg/kg glutamine for 5 consecutive days following JEV infection. Our data in Fig. 6A and B revealed that glutamine supplementation prolonged the survival of mice challenged with a high viral load of JEV (1 × 10^6^ pfu) and enhanced the survival rate in mice challenged with a low viral titer (1 × 10^4^ pfu). We next assessed whether glutamine supplementation could affect viral replication or intracranial inflammation, resulting protection from JEV-induced encephalitis. Spleens and brains from mice that were Mock-treated with PBS or treated with glutamine following infection with a low-dose of JEV were collected at 5 days post-infection for viral mRNA level determining. Compared with the PBS-treated group, glutamine supplementation effectively reduced viral levels in the spleens of mice (Fig. 6C). However, despite the lower viral levels in brains of glutamine-treated mice than in PBS-treated mice, the difference was not statistically significant (Fig. 6D).

Given that there was no statistical difference in intracranial viral titers, we further evaluated the level of inflammation in mouse brain by hematoxylin and eosin (H&E) staining analysis. Representative images of the morphological changes in different brain regions are presented in Fig. 6E. Both PBS- and glutamine-treated mice displayed varying degrees of inflammation and injury compared with healthy control mice. However, JEV-induced encephalitis in the glutamine-treated group was notably milder than that in the PBS-treated group, suggesting that glutamine supplementation attenuated JE.

Taken together, glutamine supplementation reduces peripheral viral titer, alleviates intracranial inflammation, and enhances the survival of JEV-infected mice.

## Discussion

Here we employed metabolomics approach to delineate the overview of intracranial metabolism mice infected with JEV and found that the metabolic profiles in the mouse brain were profoundly altered during the development and the progression of JE. In particular, the aberrant glutamine-glutamate-centered metabolism appeared to be closely associated with the establishment of JEV infection and the progression of JEV-induced encephalitis.

In general, glutamine-glutamate metabolism serves a multitude of roles in the mammalian brain. These include: (1) contributing to a pool of amino acids and building block of proteins; (2) linking to cellular energy metabolism via the TCA cycle intermediate α-KG; (3) forming GSH with glutamine-derived glutamate, cysteine and glycine, which in turn scavenge cellular reactive oxygen species and maintain redox homeostasis; and (4) maintaining a proper balance between the excitatory neurotransmitter glutamate and the inhibitory neurotransmitter GABA [32]. As a central pathway connecting energy metabolism, neurotransmitter synthesis and oxidative stress balance, glutamine-glutamate metabolism is fundamentally important for physiological brain functions. In this view, disturbances of glutamine-glutamate metabolism are associated with different brain disorders, which are best recognized in epilepsy [33], hyperammonemia [34] and depression [35].

In this study, we found that the glutamine-glutamate associated metabolism was extensively reprogrammed during JEV infection and its pathogenesis. Intracerebral glutamine was notably increased in the Early JE due to the up-regulated uptake. As mentioned in other studies, excessive accumulation of glutamine leads to increased intracellular osmolarity, thus causing astrocytic swelling and brain edema [36]. Accordingly, the high concentration of glutamine in the brain of JEV-infected mice observed presently might be detrimental for the normal physiological function of the brain at early stage of JE. Since the influx of glutamine across BBB is relatively modest, the majority of CNS glutamine is synthesized endogenously by astrocytes specifically (the amidation of glutamate to glutamine by glutamine synthetase) [37]. Our additional experiments indicate that the metabolic activity of glutamine uptake, utilization, and synthesis by astrocytes was upregulated with minimal JEV replication. However, decreased with extensive viral replication. This astrocyte dysfunction might contribute to the reduced cerebral concentrations of glutamine in advanced JE.

Abnormal glutamine-glutamate metabolism has multifaceted effects on the central nervous system. In terms of the physiological functions served by metabolites, glutamine is a common metabolic precursor for the biosynthesis of both the excitatory neurotransmitter glutamate and the inhibitory neurotransmitter GABA. The relationship between glutamine, glutamate, and GABA is often defined as the glutamine-glutamate/GABA cycle, which is a well-known concept involving the transfer of glutamine from astrocytes to neurons and neurotransmitters glutamate or GABA from neurons to astrocytes [38, 39]. This glutamine-glutamate/GABA cycle is a highly compartmentalized and tightly regulated intercellular process to ensure efficient extracellular clearance and replenishment of neurotransmitters. However, this physiological homeostasis is usually disrupted under disease conditions, such as intracranial inflammation [40], traumatic brain injury [41, 42], Alzheimer’s disease [43], epilepsy [33], and others. In the context of JEV infection, different steps of glutamatergic transmission were shown to be targeted in both in vitro and in vivo studies [44]. A study performed JEV intracerebral infection in 12-day-old rats and found increased glutamate levels in brain [45]. However, this is contrary to our results. We speculate that this discrepancy may be due to differences in the subjects chosen and the infection methods used. In addition, reduced expression of GLS, ionotropic and metabotropic receptors, and glial glutamate transporters were also found after JEV infection in adult mice [46]. This is consistent with our results that the expression level of GLS was significantly down-regulated after JE occurence. Our study reveals that even though glutamine levels rise significantly at Early JE, the production of glutamate does not increase due to the suppression of GLS. Although the intracerebral glutamate was not significantly altered in Early JE group, its level decreased sharply with the reduction of glutamine at advanced stage. This low activity of glutamate synthesis and glutamatergic transmission may reflect a compensatory mechanism to avoid excessive excitotoxicity induced by glutamate [44]. Furthermore, microglia infected with JEV exhibit high level of glutamate and TNF-α, thereby promoting the excitotoxic neuronal death and exacerbating encephalitis [47]. This suggests that glutamate, as an excitatory neurotransmitter, may play a complex role in the context of JEV infection.

In contrast to the metabolic reprogramming pattern of glutamate, the level of GABA was notably up-regulated at Early JE, and was slightly reduced at Advanced JE. Thus, the alteration of GABA-glutamate equilibrium resulted in a sustained high GABA/glutamate ratio in JEV-infected brain, which is secondary to the aberrant glutamine-glutamate metabolism described before. We next determined the metabolic pattern of glutamate and GABA in Early-JE by brain immunofluorescence. It is noted that the abundance of GABA is markedly enhanced in the microenvironment of viral infection, while the level of glutamate is reduced. This metabolic pattern were consistent with JEV-induced neuronal metabolic reprogramming in vitro, in which viral infection promoted the expression of GAD67, resulting in a high GABA/glutamate ratio. Furthermore, the exogenous addition of GABA also favored JEV propagation in Neuro2a cells, owing to the immunosuppressive effect of GABA on neurons.

Different types of neurons exhibit different susceptibilities to pathogen infections due to their unique innate immune programs and gene expression patterns. In a study of West Nile virus (WNV) infection, it was found that cerebellar granule cell neurons (GCNs) had higher levels of type I interferon-induced gene expression compared to cortical and Purkinje neurons, thus exhibiting greater resistance to viral infections [48]. Zika virus (ZIKV), on the other hand, preferentially targets neural progenitor cells (NPCs), leading to impaired fetal brain development [49]. SARS-CoV-2 selectively infects dopaminergic neurons, causing inflammation and cellular senescence [50]. Recently, a single-cell sequencing study on JEV-infected mice also focused on the differential susceptibility of various neuronal types to JEV infection [51]. The study indicated that JEV is susceptible to both glutamatergic and GABAergic neurons in the brain, with a slightly higher susceptibility in GABAergic neurons compared to glutamatergic neurons. Therefore, the intrinsiccellular environment of different types of neurons leads to different tropisms for viral infections, which is of great significance for elucidating the pathogenic mechanisms of viruses. Our data presented here suggested that a high GABA/glutamate ratio can suppress neuronal immunity, thereby providing a favorable environment for viral replication. This also implies that there may be differences in susceptibility to JEV infection between glutamatergic and GABAergic neurons. However, further studies are required to delve deeper into this matter.

In addition to maintaining the neurotransmitter homeostasis in the brain, another crucial role of glutamine-glutamate metabolism is participation in the synthesis of GSH, the predominant intracellular antioxidant and redox regulator. Here we describe a reduction of glutamine and glutamate in Advanced JE, thereby causing the depletion of GSH. The ratio of GSH to GSSH, which has been validated as a reliable predictor for cellular redox imbalance, was gradually decreased with the progression of JE, indicating an intensive intracranial oxidative stress induced by JEV infection. Consistent with the metabolomic data in JEV-infected mouse brain, the levels of glutamine and glutamate were also decreased in JEV-infected Neuro2a cells, which subsequently led to a reduction in the content of GSH. A previous study reported that the decreased level of GSH and the imbalance in the oxidation/antioxidation system were critical roles in brain damage in rats infected with JEV [25]. Accordingly, we speculate that the disruption of glutamine-glutamate metabolism and subsequent impaired GSH metabolism might be the metabolic factors underlying JEV-induced neuronal damage and the progression of JE.

Besides the involvement of glutamine-glutamate metabolism in neurotransmitter homeostasis and redox balance, the data presented here also revealed an association of abnormal glutamine-glutamate metabolism with energy metabolism in neurons induced by JEV infection to sustain optimal viral replication. Glutamine metabolic flux analysis revealed that the uptake of glutamine and its utilization via the TCA cycle were profoundly inhibited upon JEV infection. Moreover, this inert glutamine metabolism was accompanied with the down-regulation of GLS, thereby reducing the hydrolysis of glutamine and the generation of its derivative glutamate. Consequently, the intracellular glutamine and glutamate contents were reduced in the metabolomics analysis of JEV-infected Neuro2a cells. Since glutamine is considered as the basic amino acid for protein, nucleotide and lipid synthesis, increased glutaminolysis can be a common strategy adopted by multiple viruses to reprogram the host cell metabolism [5, 19]. However, in the present study, the activity of glutamine metabolism was significantly reduced in JEV-infected neurons, especially its utilization through the TCA cycle, as indicated by glutamine flux analysis. We speculate that the small quantity of virus infection in our experiments (MOI = 0.1) might have resulted in a low demand for molecules used to generate progeny virions, thus, leading to this observed glutamine-independent phenomenon.

Although various metabolic pathways can be manipulated by virus, the energy metabolic shift towards glycolysis, rather than OXPHOS, in response to viral infection is the predominant manifestation in host cell metabolism, as demonstrated by others and in our previous study [3, 26]. In this regard, the activity of the TCA cycle was always inhibited upon virus infection, thus creating optimal conditions for viral replication such as SARS-COV-2 [15], ZIKV [52, 53], and others. Likewise, driving the TCA cycle and promoting OXPHOS artificially are also detrimental to efficient viral replication. For example, the suppression of mouse hepatitis virus replication by metabolite-mediated TCA cycle activation has been well studied [54]. Therefore, high glycolytic activity and low TCA cycle were identified as one of the hallmarks in virus-induced host cell metabolic reprogramming. Impaired mitochondrial function induced by viral infection, partially caused by the production and accumulation of ROS, might contribute to the low TCA cycle activity [55]. In addition, the intermediates of the TCA cycle often harbors anti-inflammatory and antiviral properties [13]. The best-studied intermediate, itaconate and its derivatives, induce potent antiviral response to limit multiple virus replication, including SARS-CoV-2 replication in epithelial cells [15], influenza A virus replication in peripheral blood mononuclear cells [55], ZIKV replication in neurons [14], and others.

Here, we demonstrate that the oxidation of glutamine (the metabolic axis of glutamine-glutamate-α-KG), was inhibited in JEV-infected neurons. Exogenous addition of glutamate and α-KG, which fuels the TCA cycle by enhancing the enzymatic activity of OGDH, adverse affected JEV replication in neurons. Moreover, we conducted siRNA to selectively disturb the conversion of glutamate to α-KG (targeting GLUD1) and the utilization of α-KG in the TCA cycle (targeting OGDH). The results demonstrate that the knockdown of either GLUD1 or OGDH in JEV-infected Neuro2a cells resulted in higher levels of viral propagation. Given that the oxidative metabolism of glutamine via the glutamate-α-KG axis drives the TCA cycle and adversely affects JEV replication in neurons, we hypothesized that the low flux of glutamine-glutamate metabolism induced by JEV infection is critical for its efficient propagation.

Based on the hints that glutamine-glutamate associated metabolism played a central role in JEV infection, we further explored the efficacy of glutamine supplement in JEV-infected mice. The results indicate that glutamine supplementation can reduce the peripheral viral titer, alleviate intracranial inflammation, and enhance survival of JEV-infected mice. Glutamine supplementation has been widely used as a complementary treatment for multiple clinical illnesses [31]. A recent study reported that the use of glutamine as a nutritional supplement in patients with COVID-19 may shorten hospital stay and reduce the need for ICU care [56]. For chronic viral infections, mice infected with herpes simplex virus and treated with glutamine also exhibited higher numbers of virus-specific CD8^+^T cells, thereby enhancing the IFN-γ-associated immune response and reducing the rate of reactivation of latent HSV infection [57]. Our study also reveal that the supplementation with glutamine was beneficial for mice infected by JEV. This strategy might be a promising therapeutic approach for JEV infection.

In summary, our data highlighted that the aberrant glutamine-glutamate centered metabolism is crucial to the establishment of JEV infection and the progression of JEV-induced encephalitis. Reprogramming of glutamine-glutamate-centered metabolism induced by JEV infection has profound implications for several biological processes, including the homeostasis of neurotransmitters, balance of cellular redox, as well as energy metabolism. Furthermore, our initial attempt of glutamine supplementation in JEV-infected mice also suggests a novel therapeutic approach for viral infectious diseases.

## Electronic supplementary material

Below is the link to the electronic supplementary material.

Supplementary Material 1

## Data Availability

The data sets used and/or analyzed during the current study are available from the corresponding author on reasonable request.

## Abbreviations

BBB: Blood-brain barrier
CNS: Central nervous system
GABA: γ-aminobutyric acid
GAD67: Glutamate decarboxylase 67
GLS: Glutaminase
GLUD: Glutamate dehydrogenase
GLUT: Glucose transporter
GPx: Glutathione peroxidase
GSH: Glutathione
GSSG: Oxidized glutathione
H&E: Hematoxylin and eosin
ip: Intraperitoneal
JE: Japanese encephalitis
JEV: Japanese encephalitis virus
MOI: Multiplicity of infection
OGDH: α-Ketoglutarate dehydrogenase
OPLS-DA: Orthogonal projections to latent structures discriminant analysis
OXPHOS: Oxidative phosphorylation
PCA: Principal component analysis
ROS: Reactive oxygen species
SGLT1: Sodium dependent glucose transporter 1
TCA: Tricarboxylic acid
VIP: Variable importance in the projection
WHO: World health organization
ZIKV: Zika virus
α-KG: α-ketoglutaric acid
