# Supplementary material for: Glutamine-glutamate centered metabolism as the potential therapeutic target against Japanese encephalitis virus-induced encephalitis
Source: Cell Biosci. 2025 Jan 22;15:6. doi: 10.1186/s13578-024-01340-3 (PMC11755858; doi:10.1186/s13578-024-01340-3)
Supplement: Supplementary file 1 — Supplementary Material 1 [file 13578_2024_1340_MOESM1_ESM.docx]

**Supplementary**


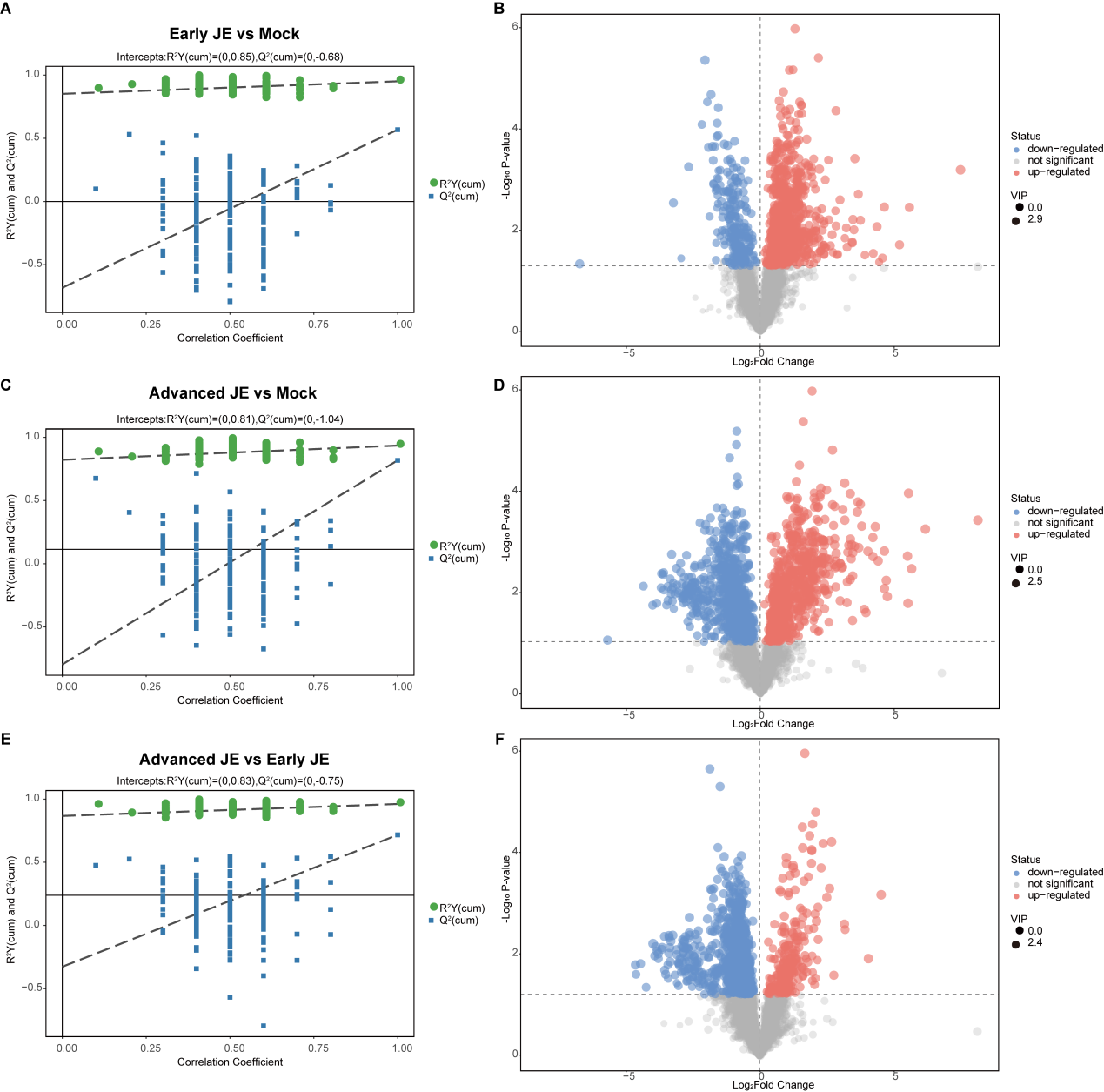


**Figure S1**

**Metabolomics analysis**

Permutation test of the untargeted metabolomics of mice brains in Early JE vs Mock (**A**), Advanced JE vs Mock (**C**), and Advanced JE vs Early JE (**E**).

Volcano plot analysis of identified differential metabolites in groups of Early JE vs Mock (**B**), Advanced JE vs Mock (**D**), and Advanced JE vs Early JE (**F**).


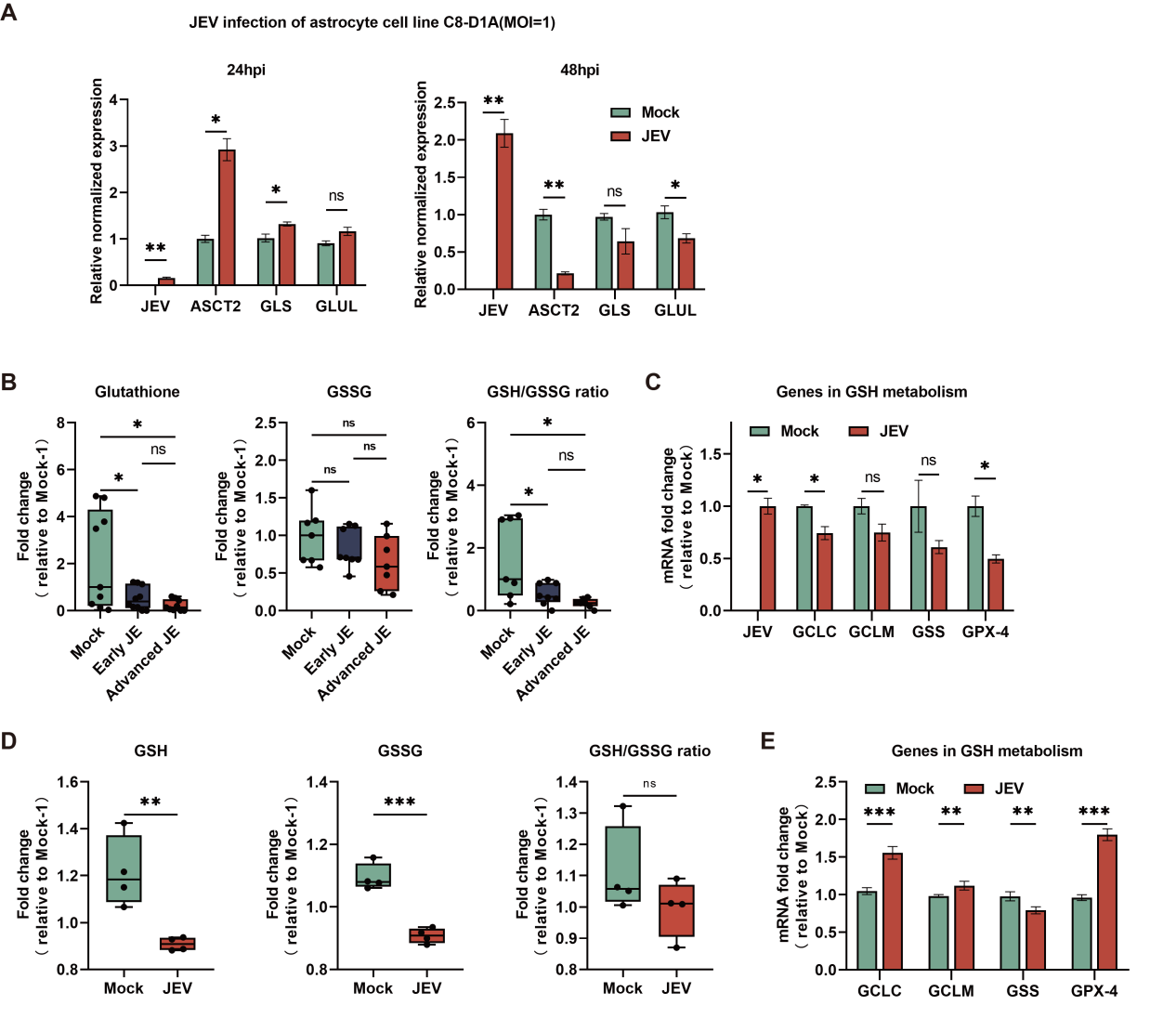


**Figure S2**

**Metabolomics analysis reveals altered GSH metabolism during JEV infection**

**A** The mRNA levels of key genes involved in glutamine metabolism in Mock- and JEV-infected murine astrocyte cell line C8-D1A. ASCT2: glutamine transporter; GLS: glutaminase; GLUL:Glutamate-Ammonia Ligase.

**B** Levels of Glutathione (GSH), GSSG and GSH/GSSG in mice brain during the development of JEV-induced encephalitis.

**C** The mRNA levels of genes involved in GSH metabolism in Mock and JEV-infected mice brains.

**D** Levels of Glutathione (GSH), GSSG and GSH/GSSG in mock and JEV-infected Neuro2a cells.

**E** The mRNA levels of genes involved in GSH metabolism in Mock and JEV-infected Neuro2a cells.


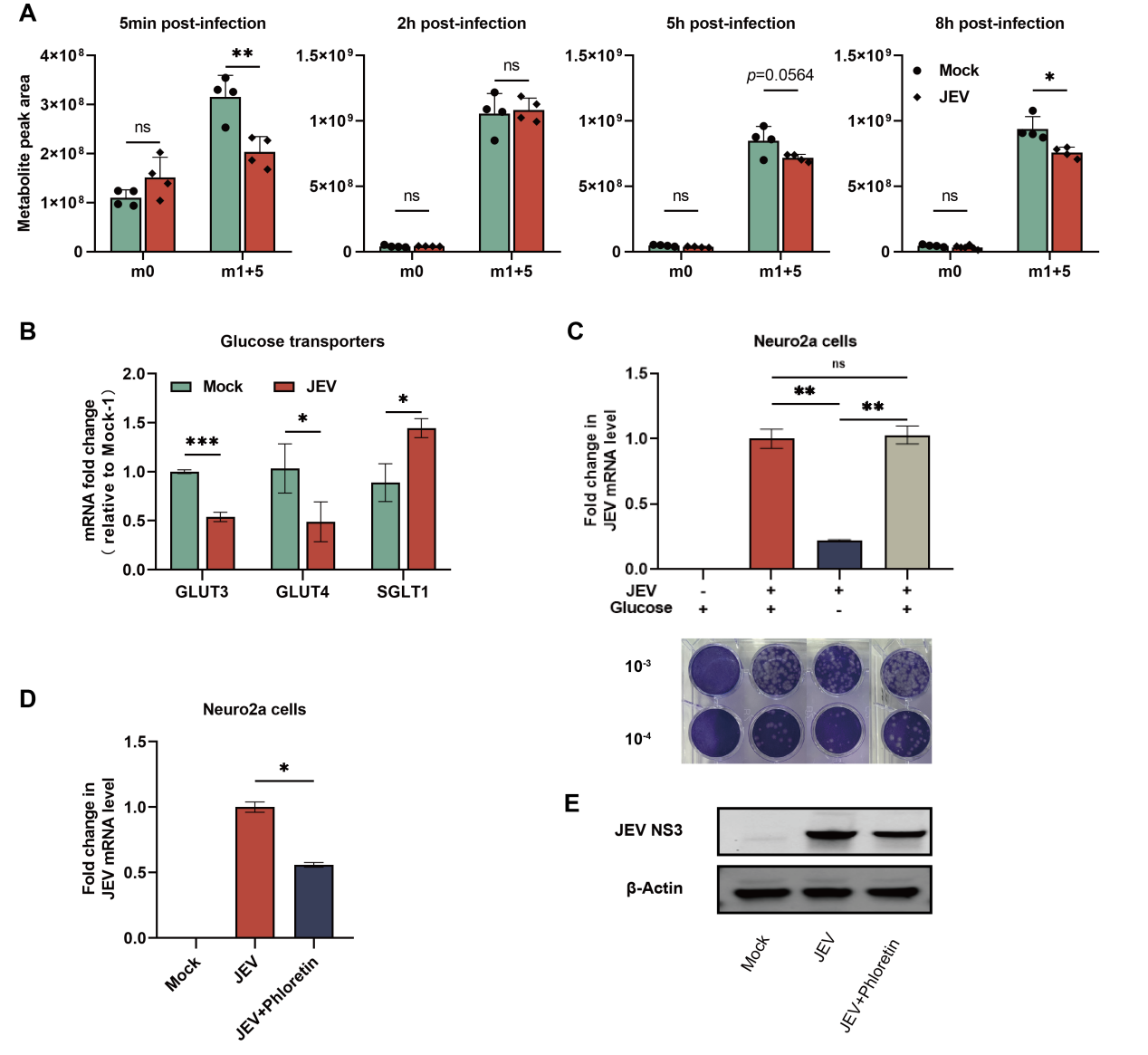


**Figure S3**

**JEV replication in neurons is more dependent on glucose rather than glutamine**

**A** Absolute quantity of isotope labeled glutamine at different time-points after viral infection.

**B** The mRNA levels of glucose transporters in Mock and JEV-infected Neuro2a cells.

**C** The level of JEV propagation in Mock- and JEV-infected Neuro2a cells treated with glucose deprivation and anaplerosis at 24 hpi were detected by qPCR and Plaque-Forming Assay.

**D** and **E** Viral mRNA levels and NS3 protein levels in Mock- and JEV-infected Neuro2a cell line treated with 20 μM Phloretin at 24 hpi were detected by qPCR and Western Blotting.

**Table S1 Primers list**

| Genes | Primer sequences（5'-3'） | |
| --- | --- | --- |
|  | sense | antisense |
| mus-GLS | AAACCTTCTGTTTGCCGCAT | TCATAATCCCGCTGCTCCAT |
| mus-GLUD1 | GTGGTCGATGTACCGTTTGG | GTCAGCGATCCAGGACATCT |
| mus-OGDH | AGGAAGGAGCTGGAGCAAAT | TGGTCTTTCCCATCACGACA |
| mus-GAD67 | AAGGGCCAATTCAGTCACCT | CTTGACCAGAATGGCAGAGC |
| mus-IFN-α | AATGGTCCTGGCAGTGATGA | GGCTCTCTTGTTCCTGAGGT |
| mus-IFN-β | CTGCCTTTGCCATCCAAGAG | TGTCTGCTGGTGGAGTTCAT |
| mus-IL-1β | AGCTCTCCACCTCAATGGAC | TTGCTTGGGATCCACACTCT |
| mus-ISG15 | AGCACAGTGATGCTAGTGGT | CCAGAACTGGTCTTCGTGGA |
| mus-TNF-α | CTGAACTTCGGGGTGATCGGT | ACGTGGGCTACAGGCTTGTCA |
| mus-GCLC | CCTGGAGCCTCTGAAGAACA | GCCAGAAGATGATCGATGCC |
| mus-GCLM | ATTGAAGCCCAGGATTGGGT | TTGGCTTGCAGAATGTAGCC |
| mus-GSS | CACGCCTGATGCTAGAGAGA | CACCTTCTTAGTCCCAGCCA |
| mus-GPX-4 | ATCTGCATGCCCGATATGCT | CCTCCCAAACTGGTTGCAG |
| mus-Actin | TGACGGGGTCACCCACACTG | AAGCTGTAGCCGCGCTCGGT |
| JEV | AGACAAGCAGATCAACCACCATT | CCCTCCAATAGAGCCAAAGTCC |
| si-mus-GLUD1 | CGGCCGAUUGACCUUCAAAUATT | UAUUUGAAGGUCAAUCGGCCGTT |
| si-mus-OGDH | GGAAAUCUCCAAGUAUGAUAATT | UUAUCAUACUUGGAGAUUUCCTT |
| si-NC | UUCUCCGAACGUGUCACGUTT | ACGUGACACGUUCGGAGAATT |
